# Femtosecond Laser-Based Integration of Nano-Membranes into Organ-on-a-Chip Systems

**DOI:** 10.3390/ma13143076

**Published:** 2020-07-10

**Authors:** Liubov Bakhchova, Linas Jonušauskas, Dovilė Andrijec, Marharyta Kurachkina, Tomas Baravykas, Alexey Eremin, Ulrike Steinmann

**Affiliations:** 1 Institute for Automation Engineering, Otto von Guericke University Magdeburg, 39106 Magdeburg, Germany; ulrike.steinmann@ovgu.de; 2 Femtika Ltd., LT-10224 Vilnius, Lithuania; linas@femtika.lt (L.J.); dovile@femtika.lt (D.A.); tomas@femtika.lt (T.B.); 3 Laser Research Center, Vilnius University, LT-10223 Vilnius, Lithuania; 4 Institute of Physics, Otto von Guericke University Magdeburg, 39106 Magdeburg, Germany; marharyta.kurachkina@ovgu.de (M.K.); alexey.eremin@ovgu.de (A.E.)

**Keywords:** femtosecond laser, microfluidic channel, organ-on-a-chip, adaptable membranes, poly-dimethyl siloxane (PDMS)

## Abstract

Organ-on-a-chip devices are gaining popularity in medical research due to the possibility of performing extremely complex living-body-resembling research in vitro. For this reason, there is a substantial drive in developing technologies capable of producing such structures in a simple and, at the same time, flexible manner. One of the primary challenges in producing organ-on-chip devices from a manufacturing standpoint is the prevalence of layer-by-layer bonding techniques, which result in limitations relating to the applicable materials and geometries and limited repeatability. In this work, we present an improved approach, using three dimensional (3D) laser lithography for the direct integration of a functional part—the membrane—into a closed-channel system. We show that it allows the freely choice of the geometry of the membrane and its integration into a complete organ-on-a-chip system. Considerations relating to sample preparation, the writing process, and the final preparation for operation are given. Overall, we consider that the broader application of 3D laser lithography in organ-on-a-chip fabrication is the next logical step in this field’s evolution.

## 1. Introduction

**Figure 1:**
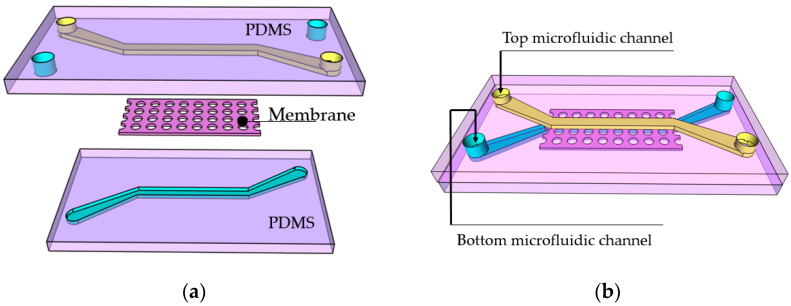
Layer-by-layer (**a**) and combined to a chip (**b**) 3D visualization of a microfluidic structure including membranes.

Organs-on-chips (OoC) are complex devices comprising a chip body, a microfluidic structure made of biocompatible transparent polymer, and a “living part”—2D- and/or 3D-cultured cells. These devices are used to study the behavior of cells and develop an understanding of their interaction. Over the past decade, numerous cell growth forms inside the channel have been reported, including micro-3D tissues [1], spheroids [2], vessels [3], and wires [4]. One of the advantages of OoC devices is the possibility to combine 2D and 3D features into a single structure. Of these, 3D cell culturing methods have their benefits, such as more extensive cell-to-cell contact interfaces, thereby reducing interactions between the cellular and extracellular environments [5]. In turn, 2D cell culture is beneficial for the study and analysis of cell–cell interfaces and barriers. Indeed, the blood–brain barrier [6,7], glomerular filtration barrier [8,9], blood-retinal barrier [10], and similar OoC applications require two or more microfluidic channels separated by a membrane [11]. The porous membrane acts not only as a support for the cells, but also has to regulate the transfer of nutrients, enzymes, hormones, vitamins and, in some cases, even bacteria between the chip compartments and allow cells to communicate. The model of a microfluidic system for such OoC applications is shown in Figure 1. There are two channels (marked in blue and yellow), that contain different types of cells. They are cultured on opposite sides of the membrane. It allows the investigation of the behavior of cells and their interaction in a well-defined closed environment. As a result, membranes have much more customized specific features than microfluidic channels and require an optimal pore size (normally in a range of 0.1–8 µm), which needs to be produced with adequate accuracy (pore size manufacturing tolerance of not more than 10%). This poses challenging requirements for the fabrication process, such as appropriate micromachining tolerances and the repeatability of structuring. Hence, OoC design dictates quite strict requirements for fabrication, in terms of the overall size of the structure (up to cm), the needed feature sizes (down to hundreds of nm), and the appropriate materials (have to be biocompatible and bioinert).

Currently, the most commonly used OoC systems are made of polydimethyl siloxane (PDMS) [12,13,14]. This material is commonly structured by molding technology, requiring a master structure produced by soft lithography. It can be used for both the channel system and membrane manufacturing. Numerous workstations and up to 20 technological steps need to be used in order to produce one OoC system using this approach. Furthermore, even with all of the successful processes, up to 40% of structures may have mechanical manufacturing failures and become unusable for the final application. This is the result of bonding. This makes the process of OoC fabrication very inflexible, tedious, time-consuming, and expensive. It is made even worse by the fact that for statistically reliable biological experiments, up to ten such systems are needed. If one needs to perform experiments with different cells of varying sizes, separate masks have to be generated and fabricated to accommodate this.

Additionally, there are commercially available membranes and filters that offer a wide range of pore sizes of 0.1–8 µm realized in polycarbonate [8,15] and polyester [9,16]. However, the pore density is fixed for each solution. The commonly proposed pore density is 105 pores/cm2 and its bonding to a chip material is challenging.

In recent years femtosecond (fs) lasers have emerged as a powerful tool for a wide range of fabrication approaches, including both subtractive and additive manufacturing [17]. In addition, multiphoton polymerization-based 3D laser lithography (3DLL) has shown huge potential in a wide array of applications [18,19,20,21]. The main advantage of this technology is the possibility to produce arbitrarily shaped 3D structures with feature size tuning from hundreds of nm to cm in the overall scale, i.e., the whole mesoscale [22]. It was also shown that structures can be produced on various functional substrates, like optical fibers or semiconductors [23,24]. Combined with an immense selection of suitable polymers [25,26,27], it shows huge flexibility and applicability. In the context of the lab-on-chip field, it was shown to be a tool capable of integrating features into channels before they are sealed [28] or even afterward [29]. The latter approach is sometimes referred to as “ship-in-a-bottle” type fabrication and could be a powerful tool for the in situ integration of functional elements into channel systems produced using other techniques. Nevertheless, while integration in glass channels is quite common in the field, working in PDMS channels and printing on PDMS, in general, are rare. This can be linked to the extremely low adhesion between most materials and PDMS [30]. On the other hand, combining 3DLL fabrication and PDMS structures is a natural progression in the field as PDMS is a viral and widespread material.

In this paper, we propose applying a femtosecond laser for the fabrication of membranes for OoC applications by printing inside a closed (i.e., assembled) PDMS microchannel. The focus of this research is directed towards fabrication and material peculiarities during such a processing regime, with the main emphasis on practical details. The main advantage of the presented approach is the possibility of varying the membranes’ size and separation easily throughout the structure without the need for additional masks or using several commercially available filters for each pore size. This enables a very high degree of flexibility for the fabrication of easily programmable 3D profiles inside a microfluidic channel. Additionally, faster iterations of organ-on-a-chip experiments can be achieved, with a wider choice of cells and bacteria that can be introduced and co-cultured in the channel. In this work, we describe the steps of the fabrication process. We address the technological challenges during processing, such as loading the polymer in the fabricated PDMS channel, optimizing the drying time and PDMS wall thickness, and optimizing the printing process parameters, development, and adhesion. We describe the considerations and discuss solutions to these challenges. Finally, we present a 1 cm long membrane integrated directly into channels of 100 and 500 µm width and a pore size of 500 nm. The technology presented in this work paves the way to the fully flexible 3D shaping of the membrane inside the channel.

## 2. Materials and Methods

**Figure 2:**
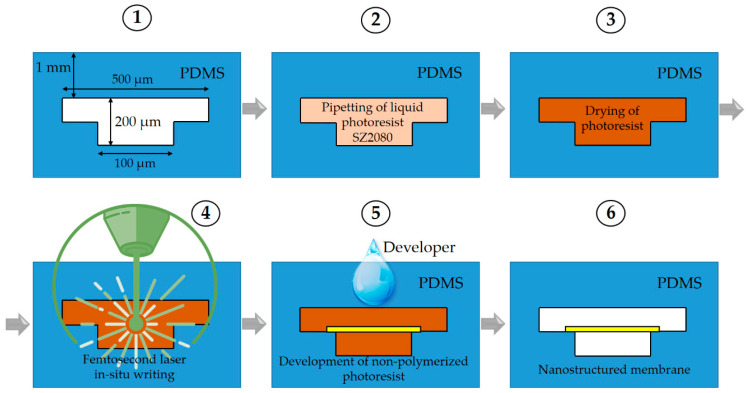
Proposed fabrication steps for printing of the microstructured membrane inside a closed microfluidic channel in polydimethylsiloxane (PDMS).

The channel systems were made out of polysiloxane (PDMS). This was chosen for several key reasons. First, it is easy to structure at a hundreds-of-micrometers scale. Second, PDMS is gas-permeable, which is necessary for cell culturing. Finally, it is transparent, mandatory for closed-channel organ-on-a-chip investigations, and allows 3DLL for direct membrane integration. The disadvantage of PDMS is its high hydrophobicity, which means it exhibits moderate adhesion properties. This can be resolved by applying chemical or plasma surface treatment. Therefore, membrane structures are stable and do not delaminate. Alternatively, off-stoichiometry thiol-ene polymer (OSTE) may be considered as an option instead of PDMS. However, with this material, it is not easy to achieve good transparency for the channel structure. This might be suitable for an open channel, but not for closed-channel applications. The fabrication steps proposed in this work are summarized in the following drawing in Figure 2. A systematic description of all the steps is given subsequently in detail in the following sub-sections.

### 2.1. Step 1—PDMS Chip Preparation

Structured fragments of PDMS (DOW Corporate, Sylgard 184) with a “sandwich structure” were prepared by molding technology. Master structures were obtained by soft lithography with the photoresist SU8-100 on a 4′ silicon wafer without an adhesion promoter. Next, the PDMS was prepared by a base to cross linker ratio of 10:1, then mixed with a magnetic stirrer (580 rpm) for 20 min. Once proper mixing was achieved, the PDMS mixture was poured onto the master structures and kept for 48 h for curing. After the fragments were cured and released, they were cut and holes were made with a 1.5 mm diameter biopsy punch. The bonding of the two structured layers of PDMS was performed by oxygen plasma surface activation over 1 min, and after alignment they was heated up to 80 °C. The different sized channels (500/100 and 1000/500 µm) were coupled to create steps where the membrane could be attached during printing.

### 2.2. Steps 2 and 3—Introducing and Drying Photoresist

The polymer used in this work was the organic-inorganic photopolymer SZ20280 with 1 of photoinitiator Irgacure 369. It is a well-established material used by multiple groups [18,31,32]. The photoresist was introduced into the microfluidic PDMS channel with a pipette. This resist was a zirconium–silicon hybrid composite, which contained both organic and inorganic networks (developed by IESL—FORTH, Heraklion, Crete, Greece). Thanks to its mechanical stability, low shrinkage, and low optical absorption for the visible spectrum, this material is an excellent choice for 3D structuring by two-photon polymerization manufacturing. Another key advantage of this photoresist is the fact that it is biocompatible, i.e., cells can proliferate on the membranes that will be generated. This photoresist has proven to be biocompatible in vitro and in vivo [31,33]. Once a homogeneous filling of the channel was achieved, the sample was heated up to 50 °C on a hot plate. The time for photoresist drying was varied in order to avoid any liquidized phases in the channel and to avoid creating air bubbles. The optimal duration was found to be 45 min.

### 2.3. Step 4—In Situ Laser Writing

Microstructuring was performed by a 3DLL technique using the “Laser Nanofactory” (Femtika Ltd., Vilnius, Lithuania) setup. We used the femtosecond laser “Carbide” (by Light Conversion), operating at the second harmonic (515 nm), with average power in the range from 0.1 to 10 mW, and a 250 fs pulse duration and repetition rate. More details on the setup can be found in [22]. Additionally, the technology is extremely adaptive for non-standard samples, such as channel systems, optical fibers, and even semiconductors. This possibility also extends to PDMS channel systems prepared previously. Writing inside close channels is possible due to the imaging system integrated into the setup as well as the possibility to use different focusing objective lenses on demand [22]. In this particular work we tested 20 × 0.4 NA (1.5 mm focusing distance, Olympus, Tokyo, Japan) and 20 × 1.4 NA (0.32 mm focusing distance, Carl Zeiss AG, Jena, Germany) lenses.

### 2.4. Steps 5 and 6—Photoresist Development

SZ2080 allows the use of different developers, including 2-propanol, 4-methyl-2-pentanone or acetone. The choice of developer is dictated by how fast the development should happen as well as what will be the capillary forces during the drying. All three were tested in this work due to different interactions with the PDMS samples. Development in 2-propanol and 4-methyl-2-pentanone was performed over 24 h, and in acetone over 8 h with continuous stirring. Afterwards, the development samples were left to dry in ambient conditions.

### 2.5. Sample Characterization

The PDMS chips and printed structures inside were characterized with an optical microscope (Olympus IX 73, Tokyo, Japan) and a confocal microscope, SP8 Confocal Laser Scanning microscope (Leica GmbH, Wetzlar, Germany). Imaging with the confocal microscope was possible without any fluorescence dye due to the autofluorescence effect of the used polymers [34]. Lines of length 552 and 488 nm were used to excite the autofluorescence of the PDMS and SZ2080, respectively. The emission was detected in the spectral range 581–695 nm.

## 3. Results and Discussion

### 3.1. Membrane Structure Geometry

**Figure 3:**
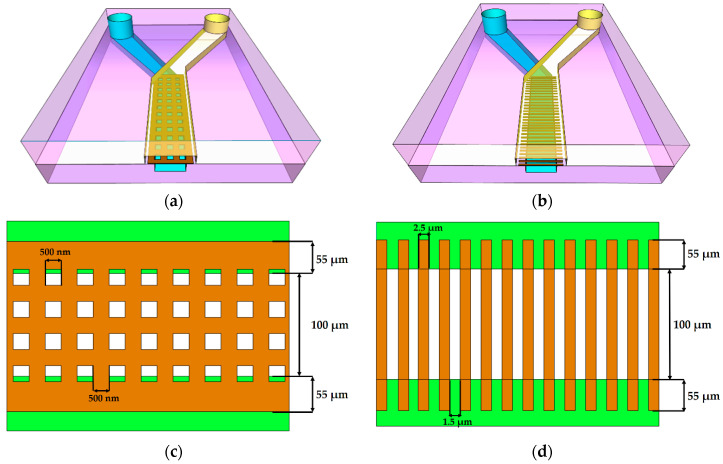
First (**a**,**c**) and second (**b**,**d**) proposed microfabricated structures, where (**a**) and (**b**) are the 3D model of the entire structure including two microfluidic channels with a square grid membrane and a wire membrane; (**c**) and (**d**) are 2D top views of the membranes with the annotated dimensions of the periodic structures.

As mentioned earlier, the primary goal of this work was to employ 3DLL for direct SZ2080 membrane integration into a PDMS channel system for OoC application. This dictates strict tolerances for the accuracy of the membranes’ fabrication and the stability and robustness of the structure. Typically, a membrane can be considered as a plate perforated periodically with a large number of holes (also termed pores) needed for communication between the cells. The openings in the membrane can have a circular or rectangular shape. The thickness of the membrane was between 5 to 10 µm depending on the objective used. A schematic of such a grid structure is given in Figure 3a,c. The width is 210 µm and the size of the square openings is 500 nm, whilst the separation, center to center, is 1 µm.

While pore arrangement is standard for OoC at the moment, from 3DLL perspective, it might limit the fabrication throughput. This is due to the necessity of making sharp turns at a sub-µm scale and/or open/close laser shutter at extremely high rates. Thus, we proposed an alternative approach shown in Figure 3b,d). The structure consisted of wires. The advantage of this structure is that one needs to write these lines instead of openings in the membrane. This means, there are considerably fewer stage movements as opposed to the first structure. Additionally, in terms of the opening between the two microfluidic channels, the effect is very similar. Hence, this structure is just as suitable for organ-on-a-chip applications, such that cells can communicate with each other. Similarly to in the first structure, the membrane width was 210 µm. At the same time, we aimed for a higher porosity of 60%, resulting in a width of 2.5 µm and the separation between the wires of 1.5 µm. These physical dimensions are optimal for the final intended application of an organ-on-a-chip. For example, epithelial cells (NCI-H292 cell line) in the stomach are about 10 µm large. Therefore, we ensured that these cells could not pass between the wires. However, proteins and nutrients, which are less than a micrometer in size, could be exchanged between the cells through these openings in the membrane.

### 3.2. Preparation for Printing

**Figure 4:**
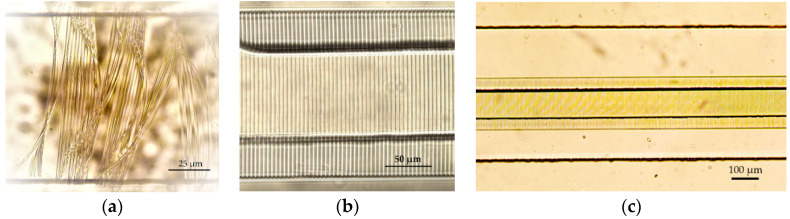
Optical images of laser-printed wire membranes for organ-on-a-chip application, where (**a**) is printed in the non-fully cured photoresist; (**b**) is a successfully printed structure in the closed microfluidic channel 100/500 µm with optimally cured photoresist and (**c**) is a 3D laser lithography (3DLL)-made wire membrane over a 1 cm long microfluidic channel.

Before 3DLL can be performed in a closed microfluidic channel, the photoresist for structuring has to be loaded and condensed inside. These two steps determine the possibility of printing and the resulting mechanical quality of the manufactured membrane. The main challenge that has to be considered at any fabrication stage in a closed channel is the appearance of gas bubbles in the channel. The presence of bubbles can result in defects in the fabricated structure due to the absence of the photoresist in the polymerization area. There are several mechanisms of how air bubbles can appear in a closed channel. First, they may appear during the loading of the liquid polymer inside the channel due to the non-uniform stream of liquid resin. Second, bubbles may also appear during the condensation of the photoresist in the channel. Therefore, the optimal time of curing needs to be determined as part of the overall process preparation. This is not an issue in standard sample preparation, as gas can freely escape from a resin drop on a glass substrate. Trying to print structures in a non-fully cured photoresist results in mechanically unstable structures, which then misalign during the fabrication process (Figure 4a). On the other hand, too much pre-baking time would result in volumetric changes of the loaded material in the channel. This leads to empty spaces, i.e., bubbles. In this case, we determined empirically that the optimal curing time was 45 min at 50 °C. This was 15 min less than the drying of photoresist in an open environment. The printed wire membrane, obtained with the care of both aspects, is shown in Figure 4b. As opposed to the structure in Figure 4a, the structure in Figure 4b is fully aligned and has a very stable, accurate repetitive separation between the wires and can be used as a periodic wire array.

**Figure 5:**
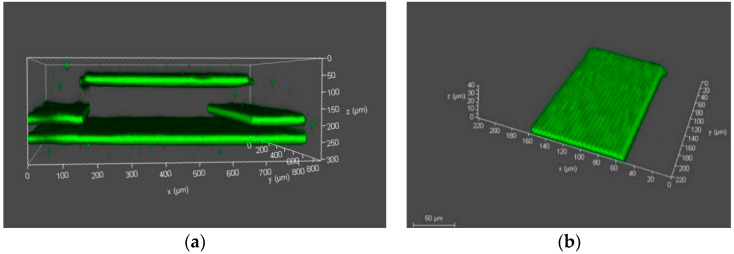
Confocal microscope images of the PDMS microfluidic channel (**a**) and printed wire membrane (**b**).

In this work, we printed the membranes to separate between two microfluidic channels over a length of 1 cm. We wanted to demonstrate the capabilities of the proposed approach using direct laser-writing. Having a longer channel length is advantageous for OoC applications, since a longer channel may accommodate more surface area for cell interactions during cell growth and culture. Figure 4c shows only part of the 1 cm structure since showing the whole structure would not allow distinguishing separate wires. During the condensation process, SZ2080 underwent a loss in volume and hardening. As a result, it deformed the PDMS carrier chip body. This means that the deformation of an otherwise even PDMS channel had to be accounted for during fabrication. Nevertheless, if condensation was performed correctly, the overall deformation rarely exceeded 5 µm. Figure 5a shows a 3D confocal microscope picture of the PDMS microfluidic channel with the deformation. The corresponding corrections of the coordinates were considered, and the printed wire membrane was very accurate, as shown in Figure 5b.

### 3.3. In Situ Direct Laser Writing

As discussed previously, printing inside a closed microfluidic channel poses several challenges. First, the choice of the PDMS wall thickness introduces a trade-off between the mechanical stability of the sample and the achievable resolution of the printed structures. This is due to the limited working distance of high-precision high-NA objectives. We conducted several experiments with different lenses to observe the pores. The PDMS thickness of fabricated chips for printing is approximately 1 mm. For the best resolution of printed structures, a 1.4 NA objective is suitable (a focusing distance of around 300 µm). However, because of the PDMS thickness (1 mm), we had to use another objective, 0.4 NA (a focusing distance of 1.5 mm). It is important to note that this also changed the transverse resolution of printing from a few hundred nm to close to 1 μm. While this did not impede the presented work, scaling laws should be kept in mind when switching objectives [22].

**Figure 6:**
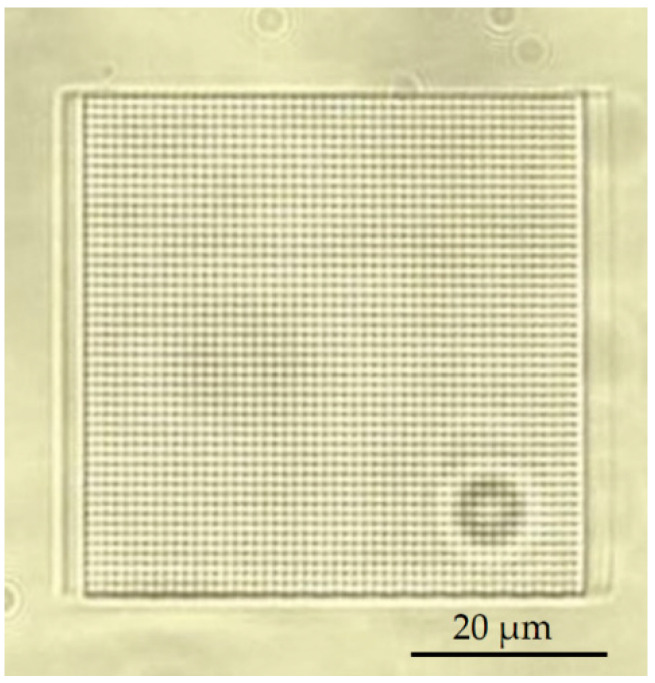
Isolated example of 3DLL-made square-grid membrane.

The next challenge of in situ printing that must be addressed is the printing time and complexity of the structure. Because the 3DLL technique writes the structure in a point-by-point fashion, for complex structures, this might take time, depending on the number of geometrical features that need to be printed. For the square-grid membrane, we realized that fabrication using the 3DLL technique was too time-consuming. The test print of the square sample (60 µm side length) is shown in Figure 6. To print over the 1 cm long channel-width structure with the grid structure takes 13 h. Due to the excessively long fabrication time and the possibility of reducing it to 7 h in the case of wires, we decided to pursue the second approach. It is important to stress that this had no negative impact on the functionality of the structure. We were able to achieve an excellent pore density by having sufficient openinga between the channels. As a final note, the printing throughput can be improved even more in the future. Indeed, by using multiplexed beams [35] or extremely fast acoustic scanning [36], throughput can be expected to improve from 10 to 100 times, making this approach even more accessible for mass implementation.

**Figure 7:**
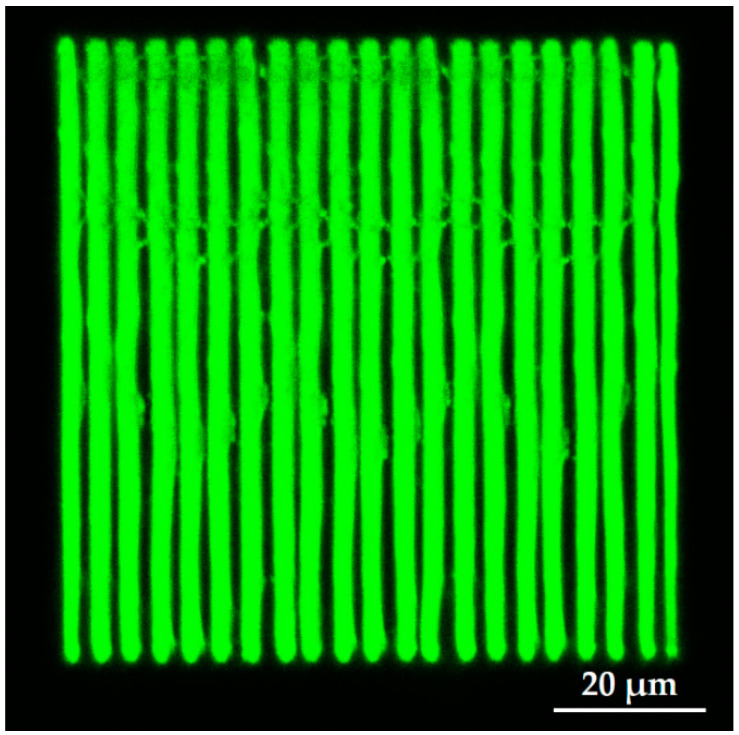
Confocal microscope image of printed wireframe with the nanoscale connections in between the microwires.

Finally, some additional challenges of printing on a large scale with a submicron resolution are the stability of the structures and possible defects. As described in the previous section, defects may appear because of the bubbles (absence of the photoresist), liquid fractions, and the physical deformation of the PDMS chip during the drying of the photoresist. Generally, the deformation can be compensated for but this leads to a longer time of the printing preparation. The unevenness of the z-coordinate needs to be compensated for. If there is an even tilting of the structure, this can be done using the built-in software functions. However, in this work, it was found that the tilt of the sample was marginal and that straightforward fabrication without any compensation could be performed. Finally, an interesting observation was made. The proposed wire membrane configuration has an additional advantage over the classical square-grid membrane since it emulates the real body membrane even better. Unintentionally, there are nanowire connections between the actually printed wires, which may act as additional support for organic substances (Figure 7). The result of the self-polymerization process occurs if two lines are made sufficiently close to each other [37].

### 3.4. Experimental Verification of Membrane

**Figure 8:**
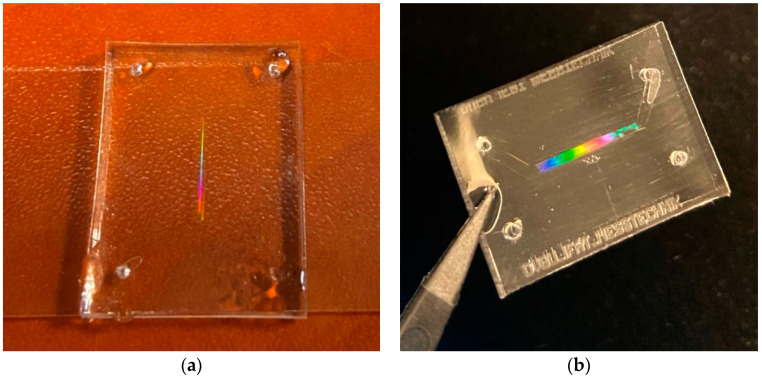
Separation of incident visible light by diffraction grating into separate colors from the fabricated PDMS structure with the 1 cm long, 100 µm (**a**) and 1mm (**b**) wide channels with micro-fabricated membranes.

To confirm by experiment that the fabricated structure was periodic and exhibited excellent microstructural accuracy properties, we considered the diffraction properties of the fabricated PDMS sample with the membrane relating to visible light. The realized wire membrane was effectively a one-dimensional periodic structure, similar to a 1D diffraction grating. According to the classical theory of Bragg reflections, such a 1D periodic structure creates the constructive interference of waves reflected from subsequent interfaces. This results in a separation of white light into the major colors of the complete spectrum, which can be observed visually under white light illumination. The mentioned effect occurs due to reflection grating, which is essentially a surface with fine grooves: in this case, pores. Figure 8 confirms this effect, as we see the separation by colors corresponding to different wavelengths. This is thanks to the periodic membrane structure, which confirms the accuracy of the fabricated periodic device.

### 3.5. Development in a Closed Microfluidic Channel

**Figure 9:**
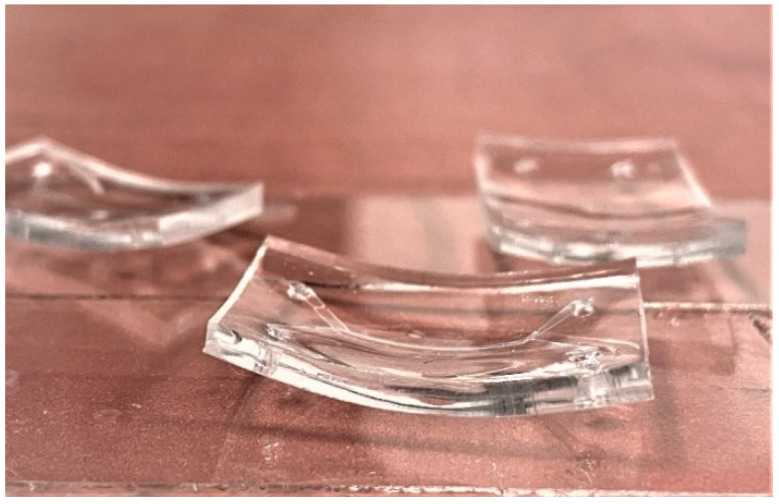
Deformation of PDMS chips in acetone and 4-methyl-2-pentanone.

**Figure 10:**
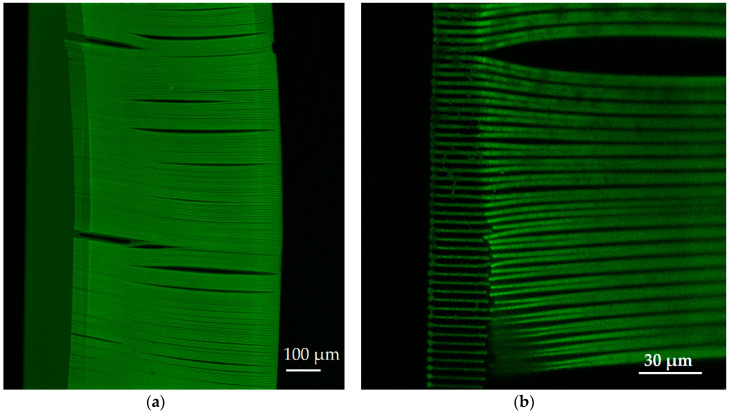
Deformation of the printed structures during development: (**a**) deformed and (**b**) cracked structure of the wire membrane.

The development of the printed structures in a closed channel using the “ship-in-a-bottle” strategy is the most challenging part of the whole process, because of the limited access of the liquid developer to the channel. The development step removes an unpolymerized substrate, thereby opening the channels completely, i.e., there is no leftover residue material which could block the channels for the cell to be introduced when needed. The organic solvent 4-methyl-2-pentanone gently develops non-polymerized photoresist SZ2080. An alternative solvent, 2-propanol, is a more aggressive option compared to 4-methyl-2-pentanone. Both materials are particularly suitable for open structures, where the liquid has free access from all directions. However, they are not suitable for development in a closed microfluidic channel. Acetone is the most aggressive solution and, therefore, able to develop the rest of the non-polymerized photoresist even with low accessibility. All solvents deform the PDMS body substantially, as noted in [38] and shown in Figure 9. The PDMS sample is bent after being introduced to a solvent. This mechanical deformation poses a significant challenge in development. It results in deformation and thereby damage to the printed structures, as exemplified in Figure 10. Luckily, in an open channel, the wire membrane still maintains its functionality and mechanical stability, since one can use the gentler solvent material 4-methyl-2-pentanone.

Deformation of the “chip body” also degrades the adhesion of the printed membrane to the PDMS. The attachment of the membrane to the sides of the bottom 100 µm channel was performed only by locating the focusing spot directly on the surface. Without constant moving and swelling of the polymer in the developer, such adhesion would be satisfying. In any case, the improvement of the adhesion forces between SZ2080 and PDMS is needed. One possible and most straightforward solution is to fix the PDMS chip physically on the glass to prevent deformation. Alternatively, one can consider treating the inner surface of the channel chemically using (3-aminopropyl)triethoxysilane. Then, according to our experience, the adhesion of structures is better and can hold better even despite deformation.

Another solution would be the printing of the whole microfluidic system using 3DLL. In this case, several considerations have to be made. First, the whole system has to be made out of gas-permitting, transparent elastomer. Also, the overall size in cm would mean that the volume that has to be fabricated is in the mm^3^–cm^3^ range. At the moment, elastomers suitable for 3DLL are somewhat lacking, with limited fabrication throughput [39] and/or a tendency to shrink substantially after fabrication [40]. Nevertheless, it is a recognized challenge with novel photo-curable elastomers being developed to combat it [41]. At the same time, 3DLL throughput can be tuned substantially. For instance, the main channel system can be made using a low NA objective and shell-type fabrication to rapidly fill all the volume while, at the same time, the membrane can be made using a high NA objective to achieve very high-resolution pores [22]. Such an approach would not only be a lot more flexible in comparison to the one presented in this study but would also allow integrating functional elements into the channel system, such as valves [42,43], during the same printing step. Thus, the results presented in this study can be considered as a foundation for further work on expanding 3DLL employment in OoC manufacturing.

## 4. Conclusions

In this work, we presented the fabrication of PDMS microfluidic channels with an integrated SZ2080 micromembrane for OoC applications. We proposed printing inside a closed microfluidic channel by means of 3DLL. Printing was generally successful in terms of resolution and printing throughput, as printing time on the cm-scale channel was only 7 h. This was achieved by optimizing the structure geometry from grid-like to wires, which also allowed an increase in porosity from 25% to 60%. One of the major challenges that need to be solved is the development of the structure after printing. After the introduction of the developer, the PDMS channels deform. In order to overcome this, one can consider using a different carrier material, such as off-stoichiometry thiol-ene polymer (OSTE). Alternatively, one can consider bonding the PDMS carrier to glass, or try to achieve better adhesion despite deformation. Overall, while we have fabricated functional samples, further research is needed to improve on the presented results, with the indicated results acting as stepping stones toward further research.
